# Crosstalk between fibroblasts and immunocytes in fibrosis: From molecular mechanisms to clinical trials

**DOI:** 10.1002/ctm2.1545

**Published:** 2024-01-24

**Authors:** Xingpeng Di, Jiawei Chen, Ya Li, Menghua Wang, Jingwen Wei, Tianyue Li, Banghua Liao, Deyi Luo

**Affiliations:** ^1^ Department of Urology and Institute of Urology West China Hospital Sichuan University Chengdu P.R. China

**Keywords:** fibroblast, immunocyte, fibrosis, therapy, molecular mechanism

## Abstract

**Background:**

The impact of fibroblasts on the immune system provides insight into the function of fibroblasts. In various tissue microenvironments, multiple fibroblast subtypes interact with immunocytes by secreting growth factors, cytokines, and chemokines, leading to wound healing, fibrosis, and escape of cancer immune surveillance. However, the specific mechanisms involved in the fibroblast‐immunocyte interaction network have not yet been fully elucidated.

**Main body and conclusion:**

Therefore, we systematically reviewed the molecular mechanisms of fibroblast‐immunocyte interactions in fibrosis, from the history of cellular evolution and cell subtype divisions to the regulatory networks between fibroblasts and immunocytes. We also discuss how these communications function in different tissue and organ statuses, as well as potential therapies targeting the reciprocal fibroblast‐immunocyte interplay in fibrosis. A comprehensive understanding of these functional cells under pathophysiological conditions and the mechanisms by which they communicate may lead to the development of effective and specific therapies targeting fibrosis.

## Graphical abstract

1. Advanced research indicates a strong correlation between fibroblasts and immunocytes in promoting fibrosis.
2. Fibroblast‐immunocyte crosstalk extensively engages in fibrosis progression of multiple organs.
3. This study shed light on further investigation of immunocyte‐fibroblast interactions in fibrosis.

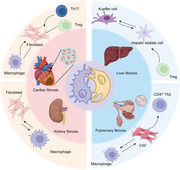

## 1 INTRODUCTION

Fibroblasts, which have been identified as ‘immune neutral’ or quiescent cells, commonly originate from stromal cells that maintain organ structure and extracellular matrix (ECM) homeostasis.^1^ Fibroblasts largely contribute to the reconstruction of the ECM in tissue repair, fibrosis and cancer. Currently, fibroblasts are no longer identified as ‘function neutral’ cells but rather as critical immune sentinel cells that trigger and regulate immune responses.^2^ For example, the immunosuppressive phenotype of fibroblasts restricts leukocyte recruitment and facilitates resolution of the immune response in mammalian skin wound healing.^3^ Moreover, neutralising fibroblast‐immunocyte interactions enhances skin regeneration.

**FIGURE 1:**
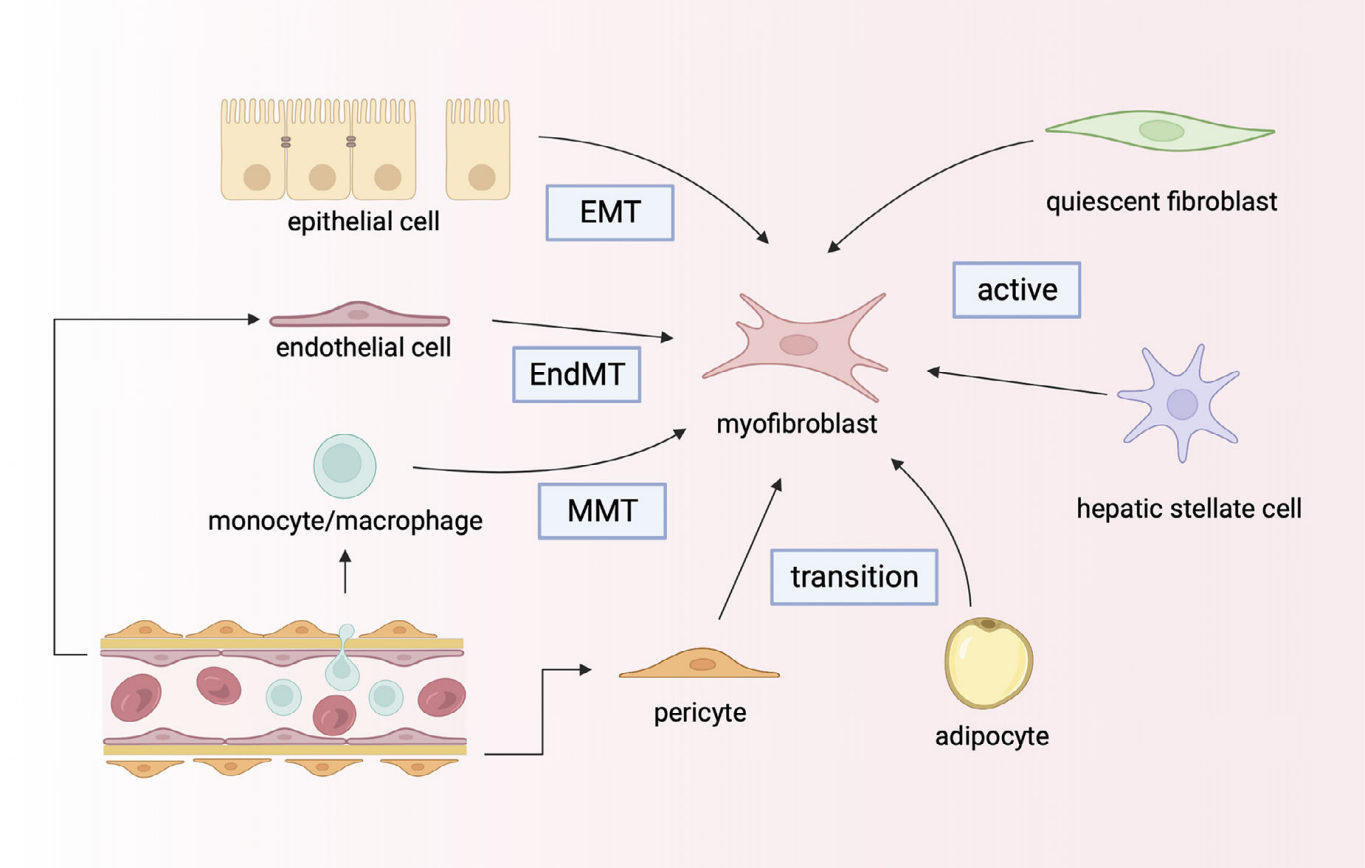
The origins of myofibroblasts.Tissue‐resident fibroblasts and quiescent stellate cells are activated and give rise to myofibroblasts. Epithelial cells are transformed into myofibroblasts through epithelial‐to‐mesenchymal transition (EMT). Endothelial cells are transformed into myofibroblasts through the endothelial‐to‐mesenchymal transition (EndMT). Monocytes/macrophages are transformed into myofibroblasts through the monocyte/macrophage‐to‐mesenchymal transition (MMT). Pericytes and adipocytes are also the precursors of myofibroblasts by transition. This figure was created using Biorender.com.

Due to the development from diverse cellular precursors, fibroblasts exhibit significant heterogeneity with various phenotypes and distinct gene patterns across multiple organs.^4^, ^5^   A cross‐organ transcriptomic profile of fibroblast lineages from 230 000 fibroblasts spanning 17 human and mouse tissues revealed distinct fibroblast‐associated gene expression patterns among the different fibroblast clusters and further confirmed the heterogeneity within the different fibroblast lineages.^6^ Tissue‐resident fibroblasts, a type of interstitial cell widely distributed throughout various tissues and organs, are generally acknowledged as quiescent or inactive fibroblasts and as major precursors of myofibroblasts (Figure 1) .^7^, ^8^ In various injured or fibrotic organs, such as the skin (dermal reticular and papillary fibroblasts),^9^, ^10^ lung (lung fibroblasts),^11^ heart (cardiac fibroblasts),^12^ liver (hepatic stellate cells, HSCs)^13^ and kidney (renal fibroblasts),^14^, ^15^ conventional tissue‐resident fibroblasts can be activated.

Increasing evidence reveals that stromal cells regulate the migration, differentiation and activation of immunocyte‐infiltrating niches. Researchers have focused on the mechanisms of fibroblast‐immunocyte interactions, including paracrine interactions, direct modulation, ECM microenvironment remodelling and mobilisation.^16^, ^17^ Furthermore, advances in single‐cell technologies have identified more distinct fibroblast subtypes under normal conditions and in disease states, providing evidence for communication between distinct fibroblast subtypes and immunocytes under various conditions. Several hypotheses have suggested that targeting fibroblasts with anti‐inflammatory mediator secretory phenotypes, which could affect immunocyte behaviour, is equally effective as targeting immunocytes in treating inflammation.^18^ Identifying the pathogenic phenotypes of fibroblast‐immunocyte interactions contributes to the interpretation of the underlying mechanisms and to the investigation of targeted therapies for fibrosis.

We systematically review the mechanisms through which fibroblasts and immunocytes interact in fibrosis and the therapeutic targets of these interactions. In addition, we also discuss the heterogeneity and origins of fibroblasts, which are highly involved in immune regulation, to highlight the therapeutic targets of pathogenic fibroblast‐immunocyte communication in fibrosis.

## 2 HISTORICAL OVERVIEW OF THE INTERACTIONS BETWEEN FIBROBLASTS AND IMMUNOCYTES IN FIBROSIS

**FIGURE 2:**
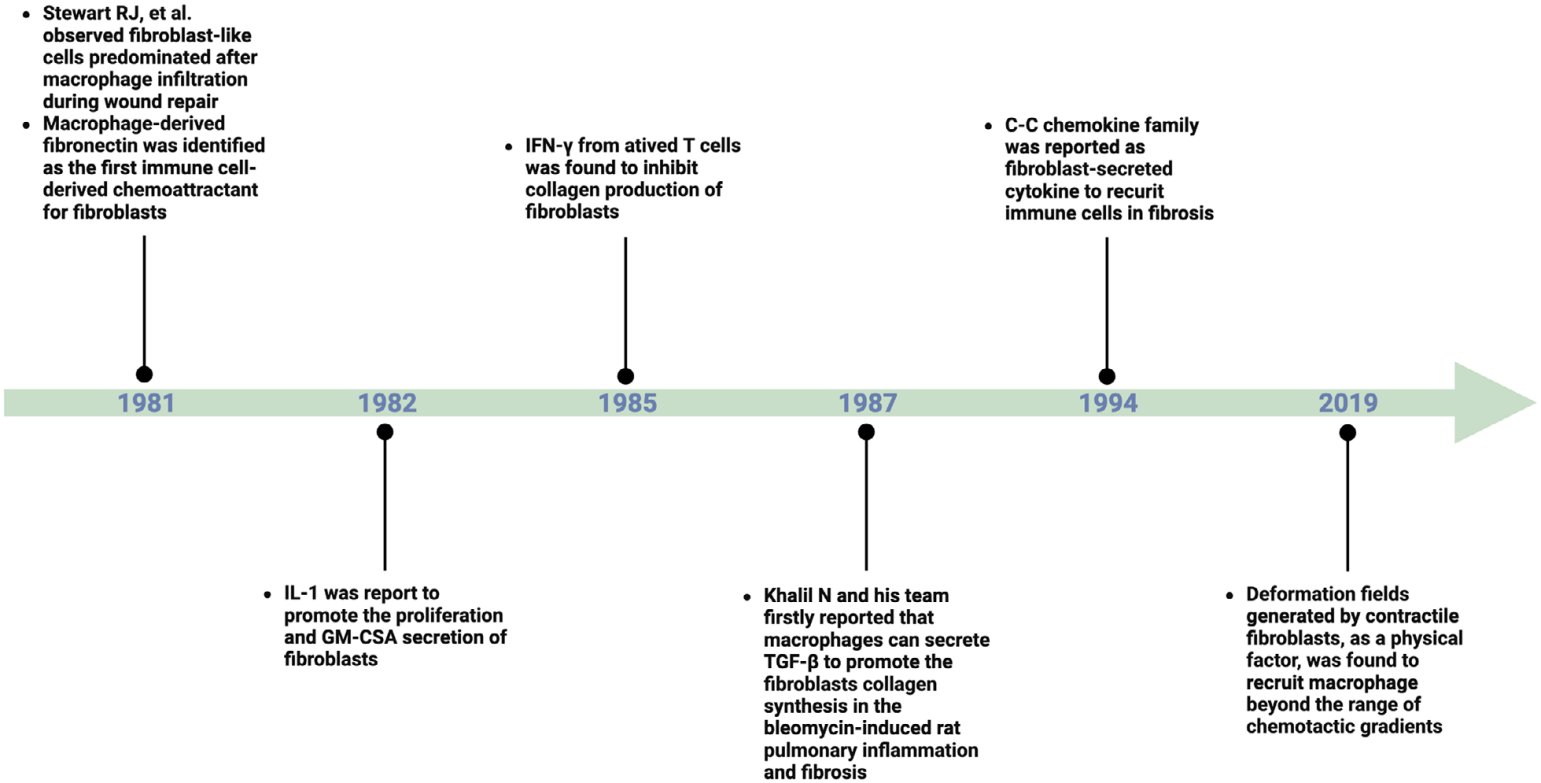
Historical landmarks in the interaction between fibroblasts and immunocytes in fibrosis. IL‐1: interleukin‐1; GM‐CSA: granulocyte‐macrophage colony‐stimulating activity; IFN‐γ: interferon‐γ; TGF‐β: transforming growth factor‐β. This figure was created using Biorender.com.

In 1981, Stewart RJ et al. reported that fibroblast‐like cells predominated after macrophage infiltration during wound repair^19^ (Figure 2). Subsequently, Tsukamoto Y et al. confirmed that macrophage‐derived fibronectin was a chemoattractant that recruits fibroblasts to the site of damaged tissue,^20^ indicating that fibronectin could be the first protein identified to mediate macrophage‐fibroblast interactions in tissue. In the early 1980s, interleukin‐1 (IL‐1) was the first interleukin found to regulate fibroblast proliferation and function,^21^, ^22^ and was confirmed to promote dermal fibroblast proliferation in the silicosis.^23^ Later, James R et al. found that IL‐1 could stimulate fibroblasts to secrete a series of cytokines essential for the maturation of granulocytes and macrophages through a process called granulocyte‐macrophage colony‐stimulating activity.^24^ Although not exhaustive, these studies provided preliminary insight into the interaction between immunocytes and fibroblasts mediated by IL‐1, both a proinflammatory and profibrotic factor, in fibrosis. Around the same time, Duncan MR and his colleagues found that interferon‐γ (IFN‐γ) produced by concanavalin A‐activated T cells could inhibit collagen production in human dermal fibroblasts,^25^ which represented the first report of the antifibrotic effect of immunocytes in vitro. Shortly afterwards, the antifibrotic effect of IFN‐γ was identified in both mice^26^ and humans.^27^

In 1989, Khalil N and colleagues first reported that macrophages can secrete transforming growth facotor‐β (TGF‐β*)*, facilitating the ECM synthesis in bleomycin‐induced rat pulmonary inflammation and fibrosis in rats.^28^ Thereafter, TGF‐β was demonstrated to be a cytokine produced by multiple immunocytes, such as regulatory B cells^29^ and phytohemagglutinin‐activated T cells,^30^ and this understanding has become one of the cornerstones of fibrosis.^31^

Since the 1990s, fibroblast‐mediated regulation of immunocytes in fibrosis has been known to involve two members of the C‐C chemokine family, C‐C motif chemokine ligand 3 (CCL3) and CCL2, which function as classical chemoattractants secreted by fibroblasts to recruit immunocytes in bleomycin‐induced pulmonary fibrosis.^32^, ^33^, ^34^ Chemotaxis via fibroblast‐derived chemokines has been widely recognised as the primary mechanism by which fibroblasts regulate immunocytes in fibrosis. In 2019, Pakshir P and colleagues found that the deformation fields generated by contractile fibroblasts, as a physical factor, can recruit macrophages beyond the range of chemotactic gradients.^35^ Although the complete mechanism by which macrophages sense deformation gradients remains unclear, this study provides a new perspective on how physical factors regulate immunocyte‐fibroblast crosstalk.

## 3 GENERAL MECHANISMS OF FIBROBLAST‐IMMUNOCYTE INTERACTIONS IN FIBROSIS

Fibroblasts are considered as the main cell type contributing to pathological fibrosis. Fibroblasts are activated by external stimuli, such as injury and inflammation, which trigger immune responses. Infectious agents are considered stimuli that can interact with pattern recognition receptors on fibroblasts activation to induce fibrosis. For example, cardiac fibrosis reduces compliance of myocardium, which contributes to diastolic dysfunction.^36^

Fibrosis is a highly orchestrated process that includes multiple cellular responses and signalling cascades.^37^ Importantly, fibrosis induces inflammatory cascades, thus recruiting immunocytes to lesions.^38^ The infiltration of immunocytes is a critical trigger of fibroblasts.^39^ The aggregation of immunocytes and fibroblasts is observed in fibrosis, indicating that fibrosis might be a complex pathophysiological process involving the participation and interaction of multiple cell types.^19^, ^40^, ^41^ For example, numerous investigations have demonstrated that fibrotic tissues can recruit inflammatory cells including macrophages and T lymphocytes.^42^ Severe or repeated tissue injury exacerbates both the innate and adaptive immune responses.^43^ Crossactivation of type I and type II immunity tries to ensure the defence and maintenance of metabolic balance. Thymic stromal lymphopoietin, eosinophils, basophils, mast cells, T helper cell 2 (Th2), group 2 innate lymphoid cells (ILC2s) and IL‐4/IL‐13‐activated macrophages are all components of type II immunity.^44^, ^45^, ^46^, ^47^ However, pathological fibrosis always leads to disturbance of this balance. The inflammatory recruitment process is sparked by IL‐1, IL‐6 and tumour necrosis factor‐α (TNF‐α), released by macrophages.^48^ Additionally, TGF‐β1, IL‐17 and IL‐18 can also enhance the proliferation of more fibroblasts.^49^ IL‐17A produces reactive oxygen species (ROS), which exacerbate tissue damage while also enhancing neutrophil responses via C‐X‐C motif chemokine ligand (CXCL)‐1/2/8.^50^ In addition, TGF‐β also serves as an accessory signal in the crosstalk between fibroblasts and immunocytes. TGF‐β1 receptors on fibroblasts are increased by IL‐17A signalling, which also leads to increased ECM secretion. Independent of TGF‐β1‐induced fibrosis, adaptive immunological CD4^+^ Th2 cells can activate fibroblasts via IL‐4 and IL‐21.^51^, ^52^ Interestingly, IL‐4 has been shown to be a powerful profibrotic cytokine that outperforms TGF‐β_,_ ^53^, ^54^ and IL‐5 produced by LC2s can also attract and activate eosinophils. Both IL‐13 and TGF‐β1 released by eosinophils can activate myofibroblasts. These interactions between fibroblasts and immunocytes largely affect the epithelial‐to‐mesenchymal transition (EMT) process in pathological fibrosis.^55^, ^56^, ^57^

In addition, single‐cell RNA‐seq (scRNA‐seq) analysis has indicated that a primordial function of fibroblasts are serving as important immune sentinel cells, which are essential for immune regulation in response to injury.^58^ Currently, additional evidence based on scRNA‐seq data suggests the interplay between fibroblasts and immunocytes in fibrosis. Herein, we summarise the mechanisms and therapeutic targets of liver, renal, pulmonary and cardiac fibrosis.

### 3.1 Fibroblast‐macrophage crosstalk in fibrosis

Macrophages are main components of natural immunity and are identified as phagocytes.^59^ Macrophages function in the clearance of debris, bacteria and foreign substances, as well as in blood‐based provisional matrix remodelling.^60^ Chemokines are the major trigger of macrophage recruitment. In the splenic red pulp, WT1^+^ fibroblasts produce the macrophage chemoattractants CCL2 and CCL7 to recruit monocytes to the open macrophage niche upon red pulp macrophage depletion.^61^ Importantly, macrophages can also recruit stromal cells, such as fibroblasts, to the ECM to initiate tissue repair and/or wound healing. Some chemoattractants, such as platelet‐derived growth facotr β (PDGFβ), are regulators of stromal cell migration and recruitment.^62^ The properties of fibroblasts and macrophages, both of which perform multiple functions in maintaining tissue homeostasis through direct crosstalk, have been studied in depth.^63^ However, interactions between these cells cannot be terminated in a timely manner in the presence of pathological stimulation, which can lead to pathological fibrosis. Hence, understanding how these cells interact with each other is of great importance in preventing pathological fibrosis.

**FIGURE 3:**
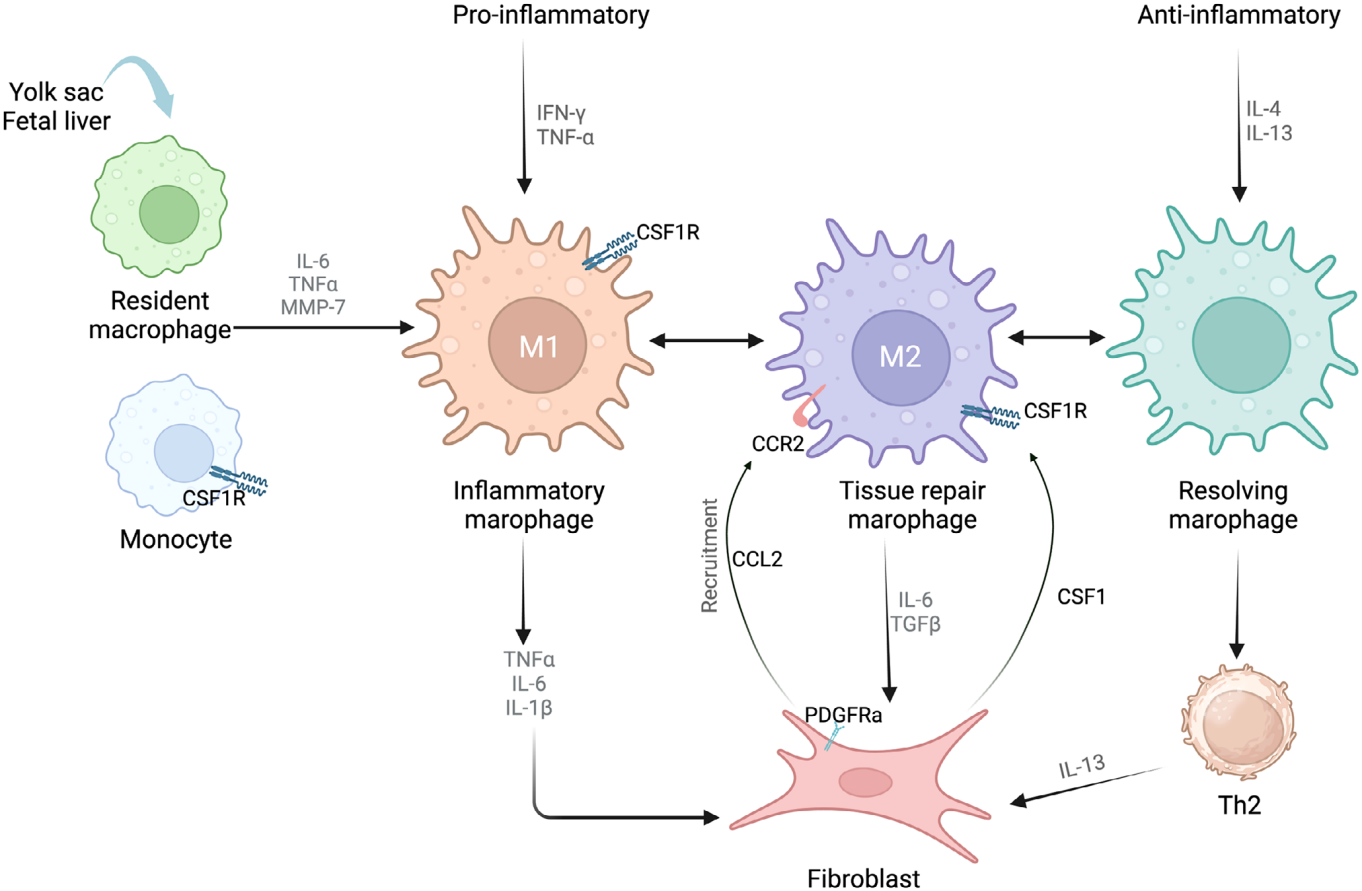
Crosstalk between fibroblasts and macrophages in fibrosis. The resident macrophages originate from the yolk sac and foetal liver. In the steady state, the macrophages are recruited by colony‐stimulating factor 1 (CSF1), or chemokines, such as C‐C motif chemokine ligand 2 (CCL2). When tissue injury occurs, the macrophages are supplied by inflammatory monocytes from bone marrow. Resident macrophages synthesise cytokines and proteins to induce macrophage proliferation, differentiation and phenotypic transformation, thereby interacting with and activating fibroblasts. Proinflammatory cytokines promote transformation of macrophages into inflammatory macrophages. Tissue repair macrophages are responsible for extracellular matrix remodelling. Resolving macrophages have an anti‐inflammatory phenotype and can secrete interleukin‐13 (IL‐13) to activate fibroblasts. Fibroblasts may secrete transforming growth factor‐β (TGF‐β) and IL‐6 to activate of macrophages. This figure was created using Biorender.com.

In response to injury stimulation, growth factors, cytokines and other mediators resident in the local microenvironment promote macrophages to facilitate tissue repair, regeneration and even fibrosis.^45^ The mechanisms by which macrophages regulate the behaviours of fibroblasts have been widely investigated in fibrotic diseases (Figure 3). Activated macrophages commonly originate from the yolk sac or from bone marrow‐derived monocytes.^64^, ^65^ M1 macrophages secrete TNF‐α, IL‐1β and IL‐6, thereby activating fibroblasts. During fibrosis progression, macrophage polarisation is facilitated, and M2 macrophages produce excessive TGF‐β, fibroblast growth factor (FGF), galectin‐3 and activin A to activate fibroblasts. Activin A, a member of the transforming growth factor family, is a critical factor preferentially released by M1 macrophages and regulates the expression of C‐C chemokine receptor type 2 (CCR2), which determines macrophage polarisation.^66^, ^67^ Activin A and CCR2, which are proinflammatory and profibrotic mediators, promote fibrosis by modulating macrophage function.^68^ Researchers applied the CD11b‐DTR system in mice to deplete macrophages at the initiation of hepatic fibrosis, and the results suggested that fibrotic injury was alleviated in this model.^69^ Moreover, loss of macrophages prolonged fibrosis progression.

In general, colony‐stimulating factor 1 (CSF1) functions in macrophage survival differentiation and proliferation.^70^ Fibroblasts maintain *Csf1* expression in both steady and fibrotic states,^71^ which triggers the chemotaxis of resident macrophages and newly migrated macrophages in the fibrotic niche. For example, Yes associated protein (YAP), the critical downstream molecule of the Hippo signalling pathway, directly modulates *Csf1* expression in fibroblasts.^72^ In fibroblasts, activated YAP enhances the expression of *Csf1*, which can recruit and facilitate the proliferation and migration of macrophages. Additionally, myofibroblasts synthesise CCL2, indicating that activated fibroblasts also recruit monocytes/macrophages to fibrotic lesions.^73^

Mechanistically, fibroblasts express the class III protein tyrosine kinase receptor PDGF receptor α (PDGFRα), which binds to mitogens of the PDGF family.^71^ Macrophages may participate in fibrosis through the expression of PDGFs. Moreover, scRNA‐seq analysis suggested that the major ECM‐expressing cells in the mouse kidney express *Pdgfrα*/*Pdgfrβ*.^14^ For instance, elevated *Pdgfα/β* expression was found in macrophages in human idiopathic pulmonary fibrosis (IPF)^74^ and in mouse models of pulmonary macrophages.^75^ Researchers discovered that activated macrophages promoted fibroblast migration and proliferation in vitro, which was inhibited by PDGF‐AA blockade.^75^ Furthermore, PDGFRα signalling cascades in fibroblasts serve as important stimuli in fibrosis progression.

Overall, the interactions between macrophages and fibroblasts are complex and depend mainly on the expression of CSF1 and the attractants secreted by macrophages (e.g., CCL2). IL‐6 produced by fibroblasts is essential for the activation of macrophages.^17^, ^76^ Conversely, macrophages can also synthesise TGF‐β, IL‐6 and IL‐17 to promote fibroblast activation.^77^ Of note, scRNA‐seq analysis revealed that ligand‐receptor crosstalk was driven by microRNA‐21 in fibroblast‐macrophage interactions.^78^ Importantly, macrophages can be transformed to myofibroblasts,which contribute to fibrosis. Chen and colleagues identified myofibroblasts as being transformed from macrophages through CD68 and α‐smooth muscle actin (α‐SMA).^79^ Therefore, fibroblast‐macrophage interactions are involved in injury, inflammation and fibrosis progression.

### 3.2 Fibroblast‐T lymphocyte crosstalk in fibrosis

Lymphocytes are engaged in fibrosis progression,^80^ which largely depends on TGF‐β. For example, Th1 and Th2 are downregulated by TGF‐β.^81^ In addition, scRNA‐seq of kidney regeneration and fibrosis in the immunocyte landscape suggested major changes in the number of T cells.^82^ However, the mechanisms through which T cells and fibroblasts interact in fibrosis remain unclear.

#### 3.2.1 The interaction between fibroblasts and Th cells in fibrosis

**FIGURE 4:**
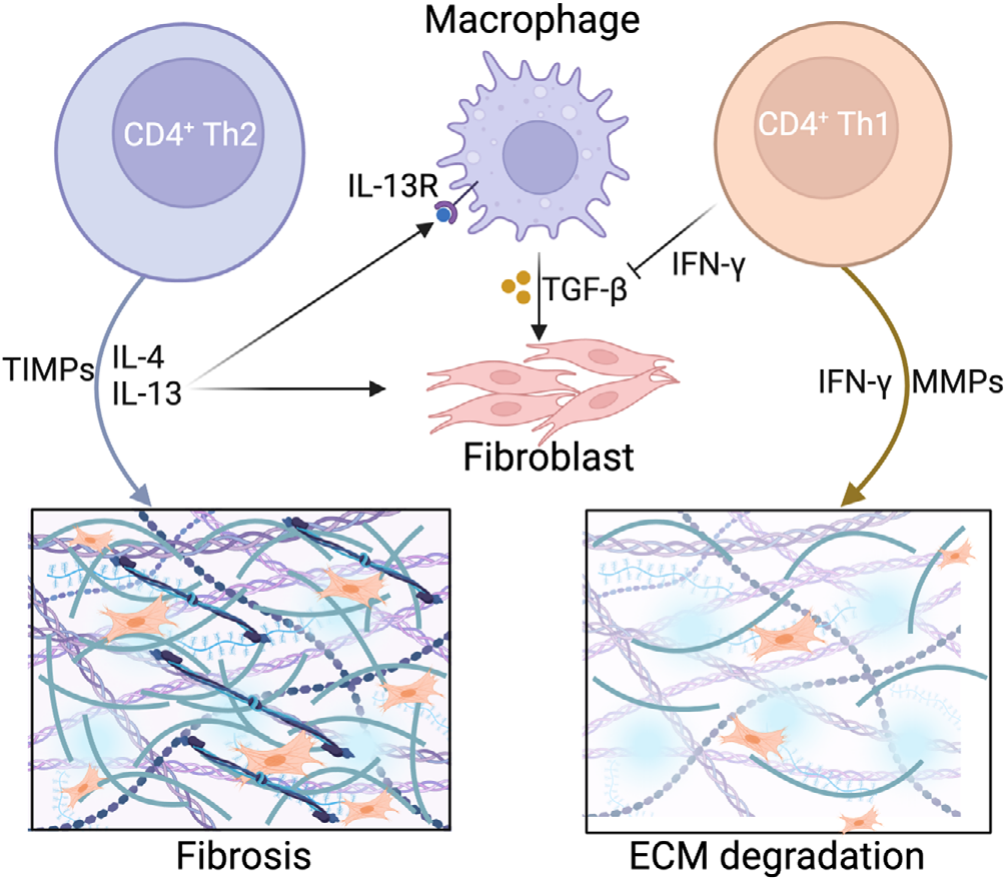
Crosstalk between T helper cell 1 (Th1) /Th2 cells and fibroblasts in fibrosis. Naïve CD4^+^cells are transformed to Th1 cells, which generate interferon‐γ (IFN‐γ), and Th2 cells to generate interleukin‐4 (IL‐4) and IL‐13. The IFN‐γ and matrix metalloproteinases (MMPs) promote the degradation of the extracellular matrix (ECM), whereas Th2 cells secrete IL‐4, IL‐13 and tissue inhibitor of metalloproteinase (TIMP) to enhance ECM synthesis. IL‐13 also combines with IL‐13 receptor (IL‐13R) on the macrophages to activate fibroblasts, which is inhibited by IFN‐γ secreted by Th1 cells. This figure was created using Biorender.com.

Intriguingly, the proinflammatory cytokines synthesised in the inflammatory process are not always profibrotic.^83^ Naïve CD4^+^ cells are transformed into Th1 cells to generate IFN‐γ (Figure 4), which can inhibit fibroblast collagen synthesis and promote the expression of matrix metalloproteinases (MMPs), such as MMP‐2, MMP‐9 and MMP‐13.^84^ Therefore, Th1 cells function in the anti‐fibrosis process.^85^ However, Th1 cells also contribute to pathological fibrosis. For example, IL‐6 induces the activation of Th1 cell effector commitment, thereby enhancing fibrosis.^86^ Moreover, in cardiac fibrosis, Th1 cells also activate cardiac fibroblasts via integrin or upregulation of TGF‐β.^80^

Th2 cells are responsible for synthesising IL‐4, IL‐5 and IL‐13. Together with Th2 cells, eosinophils, basophils, macrophages and ILC2s are all invloved in type II immunity‐induced fibrosis.^44^ For example, overactivation of IL‐4 and IL‐13 are important mediators of skin fibrosis.^87^ Rosenlum and colleagues found that Th2 cells can interact with fibroblasts in the skin fascia until adulthood.^88^ The Th2 cell niche in the skin is associated with fibroblasts, such as eosinophilic fasciitis, which is characterised by fibrosis in skin fascia. IL‐4 and IL‐13 are also essential for the initiation and maintenance of pulmonary fibrosis. High IL‐4 receptor and IL‐13 receptor expression on fibroblasts contributes to idiopathic interstitial pneumonia, indicating that these receptors may serve as potential therapeutic targets.^89^ In addition, Th2 cells induce fibroblasts to synthesise chemokines with profibrotic and proangiogenic properties in systemic sclerosis.^90^

#### 3.2.2 The interaction between fibroblasts and regulatory T lymphocytes (Tregs) in fibrosis

Tregs are another major immunocyte type that contribute to tissue repair. However, the mechanisms involved in the crosstalk between Tregs and fibroblasts in fibrosis are still largely unknown. Among CD4^+^ T cells, Tregs are the major origin of latent TGF‐β1. Specifically, Tregs can activate TGF‐β1 through integrins. Conversely, TGF‐β1 is also pivotal in the differentiation of other T cell subsets, including Th17 cells.^91^ In pulmonary fibrosis, Tregs can promote epithelial repair and inhibit the recruitment of fibroblasts, thereby suppressing fibrosis progression.^92^ Additionally, CD4^+^Foxp3^+^ Tregs are recruited in response to the presence of silica (SiO_2_) particles in the lung,^93^ where they promote the proliferation of fibroblasts through TGF‐β autocrine signalling pathway‐induced increase in the expression of PDGFβ, resulting in pulmonary fibrosis.^94^, ^95^

## 4 FIBROBLAST‐IMMUNOCYTE INTERACTIONS AND ORGAN FIBROSIS

### 4.1 Pulmonary fibrosis

Pulmonary fibrosis (PF) is one of the most prevalent lung conditions and is characterised by excessive deposition of ECM in alveoli or the lung interstitium. Due to chronic inflammation and abnormal ECM deposition, there is progressive destruction of the lung structures responsible for respiration, leading to a decline in lung function and ultimately resulting in respiratory failure and a cascade of complications.^11^, ^96^ The prevalence of PF exhibited significant regional variation. In the United States, the incidence of PF has increased. The estimated incidence of such cases among individuals aged over 65 years increased from 202 to 495 per 100 000 between 2001 and 2011, with a median survival time ranging from 2.5 to 3.5 years.^97^, ^98^, ^99^ In the United Kingdom, between 2011 and 2016, an estimated 343 million patients were diagnosed with PF, exhibiting a 7.71 (7.62−7.81) age‐standardised mortality rate per 100 000 individuals annually.^100^ Owing to the outbreak of COVID‐19, the mortality rate of PF patients witnessed a significant increase of 72% between March and April 2020 in Italy.^101^

By using genetic lineage tracing, a potent methodology that exploits genetic markers to track the developmental origin and fate of specific cell lineages in embryonic development and disease progression,^102^ Rock JR and colleagues found that, through EMT in bleomycin‐induced mouse pulmonary fibrosis, type II alveolar cells marked by *SFPTC* were not a major source of α‐SMA‐ and S100a4‐positive myofibroblasts.^103^ These results were further confirmed by DeMaio L et al., who discovered that only approximately 4% of *Nkx2.1‐*marked type II alveolar cells coexpressed α‐SMA and vimentin.^104^

**FIGURE 5:**
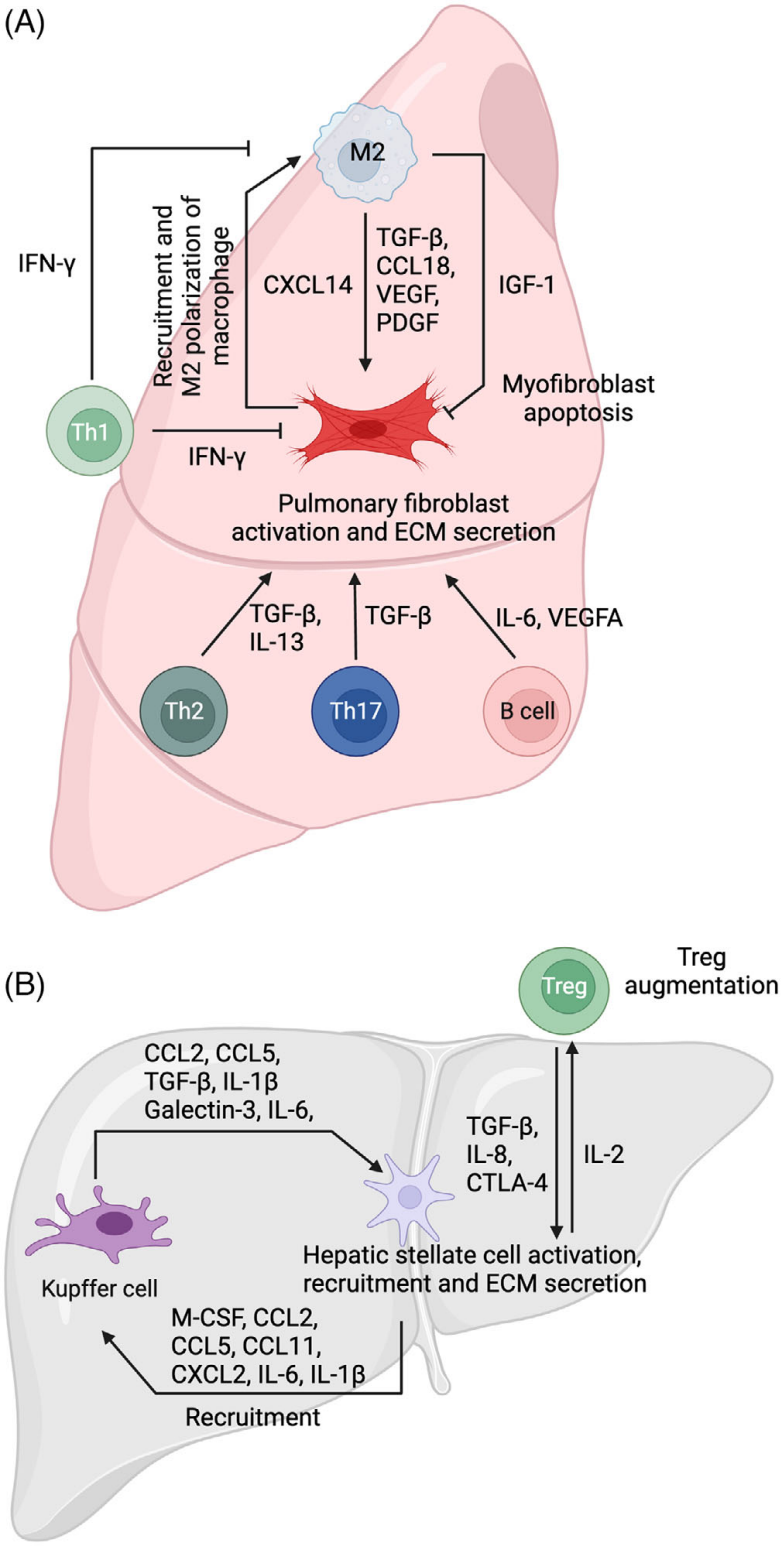
Crosstalk between immune cells and fibroblasts in pulmonary (A) and liver (B) fibrosis. In pulmonary fibrosis, M2 macrophages can secrete transforming growth factor‐β (TGF‐β), C‐C motif chemokine ligand 18 (CCL18), vascular endothelial growth factor (VEGF) and platelet‐derived growth facotr (PDGF) to promote pulmonary fibroblast activation and ECM secretion, secrete insulin like growth factor‐2 (IGF‐2) to inhibit myofibroblast apoptosis. Fibroblasts can secrete C‐X‐C motif chemokine ligand 14 (CXCL14) to recruit macrophages and make them M2 polarisation. The interferon‐γ (IFN‐γ) derived from T helper cell 1 (Th1) cells can impede M2 polarisation of macrophages and activation of fibroblasts. TGF‐β, interleukin‐13 (IL‐13) derived from Th2 cells, TGF‐β derived from Th17 cells and IL‐6, VEGFA from B cells can promote pulmonary fibroblast activation and extracellular matrix (ECM) secretion. In the liver fibrosis, Kupffer cells can secrete CCL2, CCL5, TGF‐β, Galectin‐3, IL‐6 and IL‐1β to promote hepatic stellate cells activation and ECM secretion. Hepatic stellate cells can secrete M‐CSF, CCL2, CCL5, CCL11, CXCL2, IL‐6 and IL‐1β to recruit Kupffer cells. Treg cells can secrete TGF‐β, IL‐8 and cytotoxic T lymphocyte associated antigen‐4 (CTLA‐4) to promote hepatic stellate cells activation. Hepatic stellate cells can make Treg augmentation via IL‐2. This figure was created using Biorender.com.

Crosstalk between fibroblasts and immunocytes has been identified as a crucial process in the development of PF (Figure 5A). The fibrotic niche, a complex microenvironment that develops during PF, encompasses fibroblasts, diverse immune cells and ECM. The interactions and signalling pathways within this niche have been confirmed to play crucial roles in the pathogenesis of PF.^105^ Among them, M2 macrophages are the main source of chemokines that recruit and regulate fibroblasts.^106^ TGF‐β, one of the most important cytokines, can induce fibroblast‐to‐myofibroblast transformation via the classical Smad pathway^107^ or via PU.1‐mediated epigenetic regulation.^108^ Previous in vitro studies have demonstrated that culture supernatants of alveolar macrophages derived from patients with IPF can induce collagen production in normal pulmonary fibroblasts via CCL18.^109^ In addition to CCL18, macrophages cansecrete various growth factors, such as vascular endothelial growth factor (VEGF) and PDGF, to activate fibroblasts,^110^, ^111^ which can be suppressed by nintedanib.^112^ The secretion of insulin like growth factor‐1 by M2 macrophages also contributes to the inhibition of myofibroblast apoptosis, which is critical for the maintenance of fibrosis.^113^ Moreover, fibroblasts can regulate macrophages in pulmonary fibrosis to form a regulatory circuit with each other to aggravate pulmonary fibrosis. In a crystalline silica‐induced mouse PF, CXCL14 derived from fibroblast CXCL14 can mediate macrophages recruitment and M2 polarisation.^114^

The regulation of PF by Tcells is intricate and multifaceted. Among them, Th cells are the predominant cell types regulating fibroblast functions. Traditionally, Th cells are dived into Th1 and Th2 cells. Th1 cells have been reported to possess antifibrotic properties due to their capacity for IFN‐γ secretion. IFN‐γ derived from Th1 cells can impede M2 polarisation of macrophages and activation of fibroblasts by upregulating the expression of Smad7 and disrupting TGF‐β signalling.^115^ In contrast, Th2 cells can promote fibrosis by secreting several profibrotic cytokines, such as IL‐13 and TGF‐β. Moreover, Th17 cells are a subset of T helper cells named for their ability to secrete IL‐17.^116^ It has been reported that IL‐17 might indirectly contribute to tissue fibrosis by inducing tissue injury. Moreover, Celada LJ et al. found that programmed cell death 1 (PD‐1)^+^ Th17 cells were the main CD4^+^ T cells that secrete TGF‐β and can activate lung fibroblasts by signal transducer and activator of transcription 3 (STAT3) pathway. Both blockade of STAT3 and blockade of PD‐1 could inhibit PF.^117^

Although the role of B cells in lung fibrosis remains controversial, several studies have demonstrated that B cells can stimulate fibroblast migration and activation by releasing inflammatory mediators.^118^ Additionally, the efficacy of anti‐B‐cell therapy targeting CD20 or B‐cell activating factor of the TNF family (BAFF) has been validated for the treatment of skin fibrotic diseases,^119^, ^120^ which suggests that targeting CD20 or BAFF could be a feasible method for treating PF.

### 4.2 Liver fibrosis

Liver fibrosis is a consequence of chronic liver injury and commonly occurs in individuals with various chronic liver diseases, including those with metabolic, toxic, viral and autoimmune aetiologies. A study of the global burden of disease revealed that approximately 112 million individuals worldwide are afflicted with compensated liver fibrosis, with an age‐standardised global prevalence of 1395 cases per 100 000.^121^ Although accurate data are currently unavailable,  approximately half of the two million patients are estimated to die annually from liver disease due to fibrosis‐related conditions^122^, with variations in mortality rates observed across regions due to disparities in sanitary conditions and underlying disease aetiologies.^123^ Between 1980 and 2010, the East Asia and Pacific region had the highest number of deaths related to cirrhosis, with approximately 328 000.^122^, ^123^ Additionally, the Middle East and North Africahad the highest proportion of regional deaths attributed to liver fibrosis, accounting for 3.5% of the total deaths.^122^, ^123^

In the normal liver, the periportal regions harbor a diverse array of immune cellsthat playcrucial roles in maintaining liver homeostasis.^124^ Kupffer cells, being the unique resident macrophages in the liver, play crucial roles in maintaining hepatic homeostasis and regulating various liver diseases, particularly liver fibrosis (Figure 5B). After liver injury, macrophages can recruit HSCs to the injured site by secreting CCL2 or CCL5, thereby facilitating the activation of HSCs through interaction with their respective CCR2 or CCR5 receptors.^125^, ^126^, ^127^, ^128^ Therefore, previous studies have demonstrated that the utilisation of dual inhibitors targeting both CCR2 and CCR5 could effectively suppress the recruitment of HSCs and attenuate fibrosis progression.^129^ In addition to recruiting HSCs, macrophages can modulate the activation of HSCs through various cytokines, including TGF‐β, galectin‐3 and IL‐6. The classical cytokine TGF‐β plays a crucial role in the progression of fibrosis by regulating hepatic stellate cell activation through nuclear translocation of the Smad complex, thereby controlling the transcription of genes related to fibrosis.^130^ Hence, the inhibition or knockdown of P300, a transcriptional coactivator that facilitates the nuclear translocation of the Smad complex, has been confirmed to alleviate TGF‐β‐induced activation of HSCs.^131^, ^132^ Similarly, various interleukins secreted by macrophages, such as IL‐6 and IL‐1β, can promote the proliferation and activation of HSCs through multiple signalling pathways, including the IL‐6‐STAT3 pathway,^133^ IL‐1β‐JNK pathway and the p38 signalling pathway.^134^ Moreover, the activated HSCs can also exert regulatory effects on the infiltration, recruitment and polarisation of monocytes/macrophages. Specifically, activated HSCs can recruit monocytes/macrophages through autocrine expression of M‐CSF, chemokines (e.g.,CCL2, CCL5, CCL11, CXCL2) and inflammatory factors (IL‐6 and IL‐1β), as well as enhancing monocyte from peripheral blood adhesion and retention by upregulating their adhesion‐related molecules VCAM1 and ICAM1.^135^, ^136^, ^137^ Additionally, activated HSCs can induce M1 polarisation of macrophages via Notch signalling pathways, which can be inhibited by inhibitors of Notch.^138^, ^139^ In addition to inducing M1 polarisation, the coculture of HSCs and macrophages revealed that HSCs can activate the p38 pathway to induce a distinct subtype of macrophages with both proinflammatory and profibrotic characteristics.^140^

In addition to macrophages, Tregsmarked by the nuclear transcription factor FoxP3, as well as cell surface proteins cytotoxic T lymphocyte associated antigen‐4 (CTLA‐4) and CD25, have also been reported to be involved in liver fibrosis.^141^ A previous study revealed that Treg cells are specifically augmented by HSCs in an IL‐2‐dependent manner in fibrotic tissue.^142^ Moreover, Tregs can secrete TGF‐β, IL‐8 and CTLA‐4 to directly activate HSCs or inhibit the suppressive effect of NK cells on HSCs.^141^ Hence, Tregs promote liver fibrosis through interactions with multiple cell types via various cytokines and pathways.

### 4.3 Cardiac fibrosis

Cardiovascular diseases (CVDs) cause approximately 31% of all deaths worldwide, and most cardiac diseases are associated with fibrosis of the heart, which ultimately leads to heart remodelling and failure.^143^, ^144^ In contrast to other organs, cardiac fibrosis manifests as two types: reactive interstitial fibrosis or replacement fibrosis.^143^ Reactive interstitial fibrosis is commonly observed in the case of left ventricular pressure overload as a response to preserving the pressure‐generating capacity. Fibrosis leads to the expansion of interstitial and perivascular spaces but does not cause cardiomyocyte loss.^145^ On the other hand, in the case of acute myocardial infarction, replacement fibrosis can be observed after myocyte death.^146^, ^147^

**FIGURE 6:**
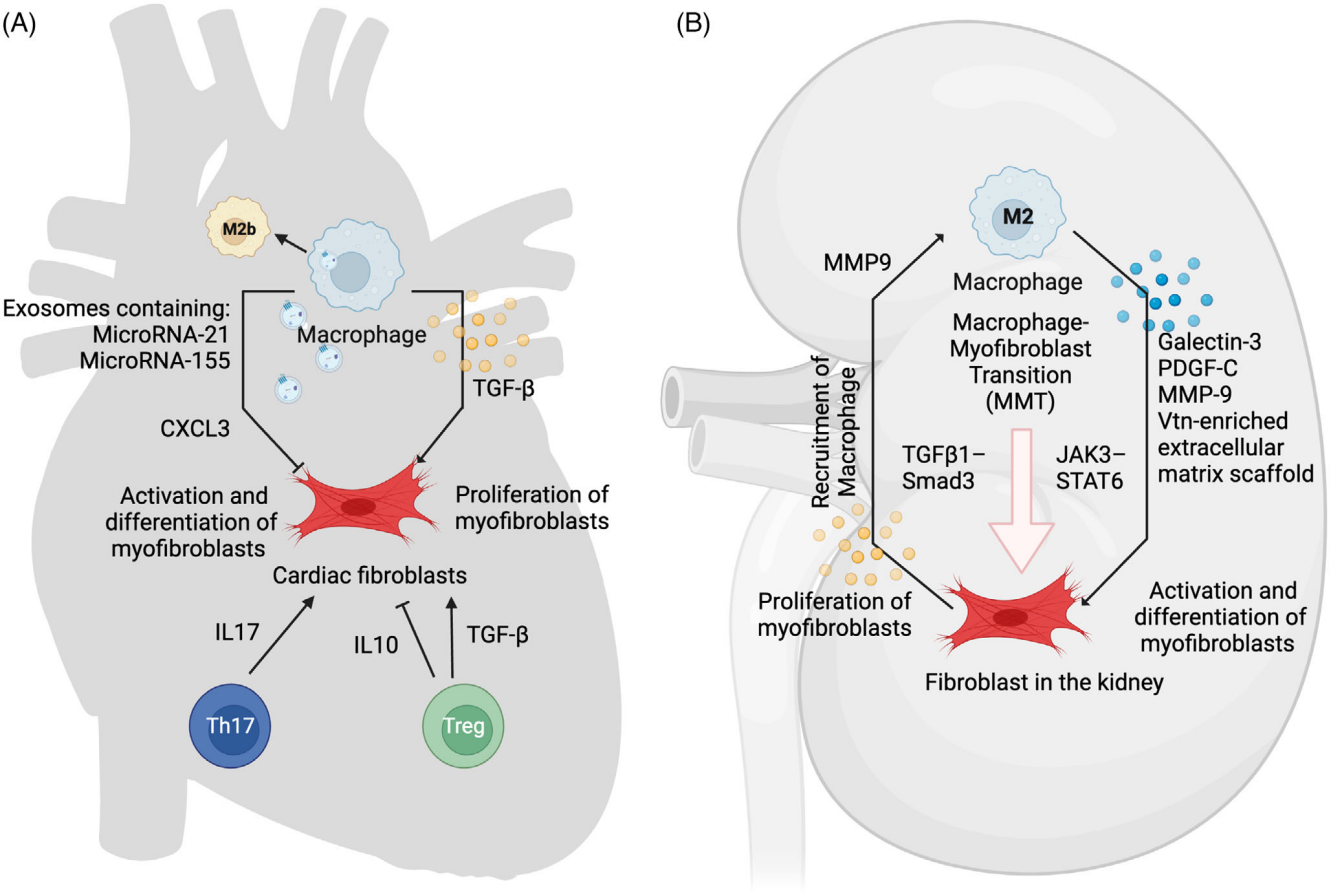
Crosstalk between immune cells and fibroblasts in cardiac (A) and kidney (B) fibrosis. In cardiac fibrosis, macrophage can secrete exosomes embedding microRNA‐21, microRNA‐155 to inhibit fibroblast activation and proliferation. It can also suppress fibroblast activity by secreting cytokines such as C‐X‐C motif chemokine ligand 3 (CXCL3). Macrophage with anti‐fibrosis property was reported to express higher level of C motif chemokine ligand 1 (CCL1) and categorised as M2b. Meanwhile, other types of macrophages may promote myofibroblast activity by secreting cytokines like transforming growth factor‐β (TGF‐β). In terms of fibroblast‐T cell interaction, increased T helper cell 17 (Th17)/Treg ratio contributes to upregulated LOX expression and fibrosis‐related indicators in fibroblasts, while a profibrosis role of Treg has also been reported via TGF‐β. In kidney fibrosis, M2 macrophage may secrete regulatory factors such as Galectin‐3, platelet‐derived growth facotr‐C (PDGF‐C), metalloproteinase‐9(MMP‐9) to promote fibroblast activation and proliferation, while fibroblast in the kidney can also promote macrophage recruitment via MMP9. In addition, macrophage can also serve as the source of myofibroblast through macrophage‐to‐mesenchymal transition, regulated by signalling such as TGFβ1‐Smad3 and JAK3‐STAT6. This figure was created using Biorender.com.

Crosstalk between myofibroblasts and macrophages plays a significant role in the process of fibrosis (Figure 6A). Macrophage‐secreted exosomes and microRNA they contain could largely impair the activity of myofibroblasts.^148^ In a left ventricle pressure overload mouse model, macrophage‐specific genetic deletion of microRNA‐21 inhibited M1 polarisation of macrophages and the related transition of quiescent fibroblasts to myofibroblasts, which protected the mouse from interstitial fibrosis and cardiac dysfunction.^78^ In another myocardial infarction mouse model, microRNA‐155 was transferred into cardiac fibroblasts by macrophage‐derived exosomes, and this transfer suppressed fibroblast proliferation by inhibiting the expression of Son of Sevenless 1.^149^ In addition to microRNA, cytokines can also mediate the interaction between macrophages and fibroblasts, and a profibrotic role of macrophages can be observed sometimes via fibroblasts. In a mouse model of heart failure in which the ejection fraction was preserved, CXCR4^+^ macrophages could augment CXCL3 expression, which promoted myofibroblast differentiation.^150^ In another study, macrophage hypoxia signalling suppressed TGF‐β1‐mediated activation of cardiac fibroblasts through extracellular signal‐regulated kinase 1/2‐dependent phosphorylation of the Smad linker region via oncostatin‐m (OSM) secretion and thus inhibit excessive fibrosis.^151^ The different roles of macrophages in communicating with fibroblasts may partly depend on their subtypes. In a recent study, M2 macrophages were further divided into M2a, M2b and M2c macrophages, which had high levels of CCL17, CCL1 and CXCL13 expression, respectively. M2b macrophages were reported to significantly suppress the proliferation, migration and activation of fibroblasts by inhibiting the mitogen‐activated protein kinase (MAPK) signalling pathway, while the opposite effects were observed for M2a macrophages in a rat model of cardiac ischemia/reperfusion.^152^ T lymphocytes are another population of immune cells that are especially important in the new therapy targeting cardiac fibrosis. An increased Th17/Treg ratio was observed in HF patients. A Th17/Treg imbalance upregulates enzyme lysyl oxidase (LOX) expression and fibrosis‐related indicators (MMP‐2/9 and collagen I/III) in human cardiac fibroblasts. Th17 cells promoted LOX expression by activating the IL‐17/ERK1/2‐AP‐1 pathway, while Tregs inhibited LOX expression by activating the IL‐10/JAK1‐STAT3 pathway.^1^ In addition, Tregs were also reported to promote cardiac fibroblast proliferation by secreting TGF‐β, while fibroblasts may also reciprocally activate Tregs.^154^ Therapy based on fibroblasts and chimeric antigen receptor (CAR)‐T cells have also been explored. Cardiac fibroblasts were found to express a xenogeneic antigen that can be effectively targeted and ablated by adoptive transfer of T cells expressing a CAR against FAP. Elimination of activated fibroblasts in a mouse model of heart disease resulted in a significant reduction in cardiac fibrosis and improved cardiac function.^155^, ^156^, ^157^

### 4.4 Kidney fibrosis

Kidney fibrosis is a pathological term in urology characteised by glomerulosclerosis, tubule atrophy, interstitial chronic inflammation and fibrogenesis, as well as vascular rarefaction.^158^ End‐stage chronic kidney disease (CKD) of any aetiology is a common outcome and final pathological change, and fibrosis ultimately leads to renal dysfunction and organ failure^159^ The prevalence of CKD G3‐G5 varies from 1.7% to 6.7% worldwide and the disease was reported to cause 35.8 million disability adjusted life years (DALYs) in 2017.^160^

Renal tissue injury and wound healing are prelusions of kidney fibrosis.^161^ Local fibroblasts and pericytes are recruited and activated after the injury, secrete regulatory molecules and synthesise ECM components.^162^ Currently, with the development of spatial transcriptomic technology, increasing evidence has indicated that kidney fibrosis start starts from the ‘fibrotic niche’, a specialised microenvironment in which sophisticated interactions between different cellular components, including mesenchymal cells, immune cells and tubular epithelial cells, etc., occur.^14^, ^15^ Along with surface markers vimentin, collagen‐1α1 (Col1a1), CD73, PDGFRβ and fibroblast‐specific protein‐1 (FSP1)/S100A4,^163^ myofibroblasts are prominent contributors in kidney fibrosis. They can not only produce ECM, but also interact actively with immune cells.

Macrophages are another important component of the fibrotic niche (Figure 6B). The accumulation of M2 macrophage, which express CD206 and/or CD163, is a common feature of active fibrotic lesions and is significantly correlated with the degree of glomerulosclerosis, interstitial fibrosis and tubular atrophy.^164^, ^165^ A retrospective study based on kidney allograft biopsy samples from 13 patients revealed that CD163^+^ M2 macrophages were frequently localised in interstitial fibrosis areas exhibiting type I collagen deposition and accumulation of α‐SMA^+^ myofibroblasts, indicating potential interplay between these two types of cells.^166^ In animal models, M2 macrophages were found to promote fibroblast proliferation and activation by secreting various molecules. In a mouse model of progressive renal fibrosis (unilateral ureteric obstruction, UUO), galectin‐3 secreted by macrophages was upregulated, and adoptive transfer of wild‐type but not galectin‐3(‐/‐) macrophages restored the fibrotic phenotype and renal myofibroblast accumulation/activation in galectin‐3(‐/‐) mice.^167^ Increased PDGF‐C expression by infiltrating macrophages was observed in fibrotic areas in a UUO model, and treatment with anti–PDGF‐C reduced interstitial myofibroblast accumulation by 57%.^168^ A macrophage‐derived, Vtn‐enriched extracellular matrix scaffold was found to promote fibroblast activation and proliferation by stimulating integrin αvβ5 and Src kinase signalling.^169^ Inhibition of (MMP‐9 produced by macrophages in late‐stage UUO reduced tubular cell EMT. Interestingly, MMP‐9 can also be expressed by myofibroblasts and further recruit macrophages.^170^ In addition, in the context of kidney fibrosis, macrophages derived from bone marrow cells can differentiate into α‐SMA^+^ myofibroblasts (termed MMT) and the bone marrow‐derived fibroblasts were reported to account for 35% of the total myofibroblasts in a UUO model.^171^, ^172^ The MMT process is regulated by a complex signaling network that includes canonical TGF‐β1–Smad3 signalling, a Src‐centric regulatory network, and the JAK3–STAT6 signalling pathway.^173^, ^174^, ^175^

## 5 CLINICAL TRIALS TARGETING FIBROBLAST‐IMMUNOCYTE INTERACTIONS IN FIBROSIS

Many therapies,including several mediators that target fibroblast‐immunocyte interactions, have been identified as critical treatments for fibrosis. In this section, we briefly focus on the typical therapeutic targets associated with fibroblast‐immunocyte interactions in fibrotic diseases based on clinical trials. We categorised these antifibrotic targets and drugs according to the accessible clinical research data in Table 1.

**TABLE 1:** Drug targets and clinical trials targeting fibroblast‐immunocyte interaction in fibrosis.

Targets		Drug name	Disease	Phase	NCT	Status	Sample size
Galectin	Galectin‐3	Belapectin	NASH cirrhosis	2b/3	NCT04365868	Recruiting	1010
	Galectin‐3	GB1211	NASH cirrhosis	2	NCT04607655	Withdrawn	0
	Galectin‐3		IPF	2	NCT03832946	Active, not recruiting	426
FGF	FGF21	BIO89‐100	NASH cirrhosis	2	NCT04048135	Active, not recruiting	101
	FGF21	Efruxifermin	NASH cirrhosis	2	NCT03976401	Completed	110
	FGF21	Pegbelfermin	NASH cirrhosis	2	NCT02413372	Completed	184
	FGF19	Aldafermin	NASH cirrhosis	2	NCT03912532	Completed	171
PDGFs/PDGFRs	PDGFRs, FGFRs, VEGFRs	Nintedanib	IPF	Marketed	NCT02598193	Completed	89
	PDGFRα, β, FGFR1‐4, and VEGFR1‐3	ZSP1603	IPF	2	NCT05119972	Recruiting	36

Abbreviations: FGF: fibroblast growth factor; FGFR: FGF receptor; NASH: nonalcoholic steatohepatitis; IPF: idiopathic pulmonary fibrosis; PDGF: platelet‐derived growth factor; PDGFR: PDGF receptor; VEGF: vascular endothelial growth factor.

### 5.1 PDGFs/PDGFRs

PDGFs, which are growth factors, are profibrotic cytokines that activate fibroblasts and the EMT process. PDGFs are expressed in macrophages, fibroblasts and endothelial cells.^74^, ^176^ Importantly, PDGFs are critical signalling molecules involved in the crosstalk between fibroblasts and immunocytes. For example, PDGF‐B and PDGF‐D are pivotal for HSC proliferation and migration, leading to ECM deposition in hepatic fibrosis.^177^, ^178^ Nintedanib, a receptor tyrosine kinase inhibitor, mainly targets growth factor signalling molecules, such as FGFRs, VEGF receptors (VEGFRs) and PDGFRs.^179^ Nintedanib alleviates pulmonary inflammation and fibrosis by degrading ECM, chemokines and profibrotic factors.^180^ Several clinical trials have demonstrated the efficacy of nintedanib on IPF with satisfactory safety and tolerability results.^181^, ^182^, ^183^, ^184^

### 5.2 Galectin

Galectins are lectins that include 10 members with a wide distribution in tissues.^185^ The biological activities of galectins are observed in various cell types and diseases, including in cancer, tissues and during inflammation, immune activation and fibrosis.^186^ In response to inflammatory reactions, galectin facilitates macrophage reprogramming into M2 macrophages.^187^ Galectin‐3 can regulate the macrophage polarisation and promote fibrosis.^188^ Importantly, galectin triggers fibroblast activation, migration and proliferation.^189^ Galectin‐3 has been identified as a critical therapeutic target for fibrotic diseases, such as IPF and nonalcoholic steatohepatitis (NASH).^190^, ^191^

### 5.3 FGF

The FGF superfamily consists of 10 main members.^192^ FGFs can facilitate dimerisation, activation and autophosphorylation of FGFRs and trigger downstream signalling cascades.^193^ However, how FGFs contribute to fibrosis is unclear. For example, FGF‐19 deficiency alleviates hepatic fibrosis in mouse models.^194^ However, FGF‐19 activation inversely attenuates cytokine production.^195^ In pulmonary fibrosis, FGFs have been identified as therapeutic targets that trigger the activation and proliferation of fibroblasts but inhibit the differentiation of myofibroblasts.^196^, ^197^ Moreover, FGF‐21 can reduce the recruitment of immunocytes and ECM accumulation in pulmonary fibrosis.^198^

Currently, clinical trials focusing on fibrosis are now examining a variety of compounds and small molecules that target immunocyte‐fibroblast interactions. The mechanisms of these therapies are different due to the sophisticated regulatory network of fibrotic diseases. Since the role of fibroblast‐immunocyte interactions in fibrosis has become a focus of research attention, more therapies targeting these mechanisms are being explored for improved efficacy and safety.

## 6 CONCLUSIONS AND PERSPECTIVES

An increasing number of patients suffer from multiorgan fibrosis across the world. Researchers and clinicians have focused on investigating the underlyingmechanisms and treatments of fibrosis. Many key regulatory molecular mechanisms have been identified in the recent decades. With the development of scRNA‐seq, more tissue‐resident cell types are classified to play roles in profibrotic pathology. In addition, several deeper mechanisms including how fibrotic cells communicate with immunocytes have also been identified. Nevertheless, strategies such as targeting the main cell types involved in fibrosis (e.g., myofibroblasts, stellate cells, epithelial cells) are the main approaches used to alleviate fibrosis. However, no effective drugs can inhibit the progression of fibrotic diseases.

Of note, investigators have focused on the drug delivery systems that target specific cell types in order to increase the efficiency and safety of drugs. For example, the extracellular vesicles (EVs) are ideal particles secreted by most of the cells. EVs can carry mRNAs, proteins, miRNAs and lipids to recipient cells with high compatibility. Currently, several drug delivery systems have been shown to have ideal efficacy and safety in clinical trials. However, these drug delivery systems are not consistent for all organs. Some delivery systems are easily concentrated in some organs, but others may not.

In conclusion, the current review summarises the history and mechanisms of crosstalk between fibroblasts and immunocytes in fibrosis (see the graphical abstract). The immunocyte‐fibroblast interplay affects fibrosis progression. Since fibrosis is always in an advanced stage after injury and cannot be easily reversed, the prevention and detection are important in future studies.

## AUTHOR CONTRIBUTIONS

D.‐Y. L. and X.‐P. D. designed and supervised the review; X.‐P. D., J.‐W. C. and Y. L. drafted the manuscript, X.‐P. D., J.‐W. C., J.‐W. W. and Y. L. generated figures and tables; D.‐Y. L., B.‐H. L.. T.‐Y. L. and M.‐H. W. performed manuscript reviewing and editing. All authors have read and approved the article.

## CONFLICT OF INTEREST STATEMENT

The authors declare that they have no competing interests.

## ETHICAL STATEMENT

Not applicable.
